# COX6B1 relieves hypoxia/reoxygenation injury of neonatal rat cardiomyocytes by regulating mitochondrial function

**DOI:** 10.1007/s10529-018-2614-4

**Published:** 2018-10-11

**Authors:** Wei Zhang, Yu Wang, Junzhe Wan, Pengbo Zhang, Fei Pei

**Affiliations:** 1 Department of Cardiovascular Surgery, The Second Affiliated Hospital of Xi’an Jiaotong University, No.157 West 5 Road, Xi’an, 710004 Shaanxi Province China; 2 Department of Anesthesiology, The Second Affiliated Hospital of Xi’an Jiaotong University, No.157 West 5 Road, Xi’an, 710004 Shaanxi Province China

**Keywords:** Cardiomyocytes, COX6B1, Hypoxia/reoxygenation injury, Mitochondria

## Abstract

**Objective:**

Mitochondrial dysfunction plays a pivotal role in various pathophysiological processes of heart. Cytochrome oxidase subunit 6B1 (COX6B1) is a subunit of cytochrome oxidase.

**Methods:**

Cardiomyocytes were isolated from neonatal SD rats (within 24 h of birth) by repeating digestion of collagenase and trypsin. COX6B1 over-expression and hypoxia/reoxygenation was conducted on neonatal rat cardiomyocytes. Cell viability, apoptosis rates, mitochondria membrane potential and mitochondrial permeabilization transition pores (mPTPs) were then determined respectively by Cell performing Counting Kit-8 (CCK-8), Annexin-V/PI assay, JC-1 assay, mPTP assay. The expression of cyto C and apoptosis-related factors were detected by RT-Qpcr and Western blot.

**Results:**

Hypoxia/reoxygenation increased apoptosis and mPTP levels, and decreased mitochondria membrane potential in I/R and I/R + EV groups. COX6B1 over-expression increased mitochondria cyto C, pro-caspase-3, pro-caspase-9 and bcl-2, while it decreased cytosol cyto C, cleaved-caspase-3, cleaved-caspase-9 and bax compared to I/R + EV group.

**Conclusion:**

COX6B1 protected cardiomyocytes from hypoxia/reoxygenation injury by reducing ROS production and cell apoptosis, during which reduction of the release of cytochrome C from mitochondria to cytosol was involved. Our study demonstrated that COX6B1 may be an candidate target gene in preventing hypoxia/reoxygenation injury of cardiomyocytes.

## Introduction

The incidence of acute myocardial infarction (AMI) is increasing annually, it has become a public health problem (Liakos and Parikh 2018; Rahman et al. 2018). The most effective therapeutic method for treating AMI is to achieve a complete reperfusion of myocardial tissue based on the opening of the coronary arteries, that is, the realization of myocardial microcirculation and reperfusion (Deng et al. 2018). Thus, preventing reperfusion injury and improving myocardial tissue reperfusion awaits to be properly solved (Asif and Morales 2018; Jakl et al. 2014). Simply restoring pericardial coronary blood flow is not sufficient in treating myocardial ischemia (Panagiotou et al. 2018). Effective cell protective methods should therefore be taken to reduce apoptosis and necrosis of myocardial cells (Sugiyama et al. 2018). Thus, a Full understanding of the mechanism of myocardial reperfusion injury is a prerequisite for exploring new treatment methods.

Mitochondrial dysfunction plays a pivotal role in various pathophysiological conditions including myocardial ischemia–reperfusion (I/R) injury (Feng et al. 2017; Kanaan and Harper 2017) in the heart. A large number of studies have shown that myocardial I/R could cause structural and functional abnormalities of mitochondria, which would lead to impairments of other organelles and cell homeostasis, and therefore further aggravate myocardial injury (Wang et al. 2015b; Maneechote et al. 2017). Myocardial injury can lead to not only the opening of a large number of mitochondrial permeabilization transition pores (mPTPs), but also the disruption of the mitochondrial outer membrane, thereby resulting in the release of cytochrome c from the mitochondrial intermembrane space and leading to increased apoptosis (Zhou et al. 2014; Wiczer et al. 2014; Kumazawa et al. 2014). Mitochondria-initiated apoptosis and necrosis are two major factors causing cell death during myocardial reperfusion (Dalla Via et al. 2014). Apoptosis can affect myocardial infarct size, and the increase of apoptosis rate will aggravate I/R damage. However, an effective inhibition of apoptosis can relieve myocardial injury (Yao et al. 2015; Deng et al. 2015; Liu 2014). Therefore, a better understanding of these complex mechanisms associated with mitochondrial dysfunction is a prerequisite for exploring novel potential therapeutic targets for maintaining mitochondrial function and cellular activity in hearts with reperfusion.

Mitochondrial disease is mainly related to Cytochrome oxidase (COX) deficiency (Popovic 2013). COX belong the cytochrome system in cellular respiration. The role of COX is to transfer the electrons of the respiratory substrate directly to molecular oxygen via cytochrome system (Kemppainen et al. 2014). Cytochrome oxidase subunit 6B1 (COX6B1) is one of two small subunits in the 6th subunit of cytochrome oxidase, and the main role of COX6B1 protein is to connect two COX monomers to form a dimer. Abnormal changes of COX6B1 affect greatly the function of cytochrome oxidase, and it could lead to the development of some diseases, for example, brain myopathy (Abdulhag 2015; Kim 2015; Massa 2008).

Thus, we determined to conduct a research on the functional mechanism of COX6B1 in myocardial I/R injury. This study provides novel targets to the treatment of I/R injury.

## Materials and methods

### Animals

Ten female breeding Sprague–Dawley (SD) rats (8–10 weeks old; 25–28 g of weight) were purchased from Beijing Vital River Company (Beijing, China), and under a shift of 12 h light/12 h dark, the rats were kept at 25 °C. The animals were bred and used under the approval of Animal Management Committee of Xi’an Jiaotong University (Approval No.: AD20172650424). After the neonatal rats were born, they (within 24 h of birth) were used for cardiomyocytes extraction. 75% ethanol was used to disinfect the skin, and the heart was taken out. After large blood vessels adhered to the surface of the heart and the atria were cut off, the heart tissues were digested using collagenase and 0.05% trypsin at 37 °C for 10 min (repeated for 5 times). The supernate was then cultured by DMEM with 10% FBS for 48 h, and cell morphology and pulsatility of obtained cardiomyocytes were observed by inverted microscope.

### Cell transfection

Recombinant plasmid of COX6B1 was constructed using pcDNA3.1 Vector (Promega, USA), and transfected to myocardial cells using transfection reagent lipofectamine 3000 (Invitrogen, Carlsbad, CA, USA), and in this way, COX6B1 over-expression cells (COX6B1 group) was formed. The empty vector was transfected to myocardial cells as negative control (EV group), while myocardial cells with non-treatment were used as control (Control group). Cells were inoculated in DMEM culture media without antibiotic. When the cells reached 70%–80% confluence, the plasmid and lipofectamine 3000 (1:2) were added into DMEM culture media without serum, and the cells were then cultured for 6 h. Next, the cells were cultured at 37 °C for 48 h in an incubator with 5% CO_2_. Cell transfection rates were detected by RT-qPCR and Western blot.

### Hypoxia/reoxygenation injury (I/R) injury of myocardial cells

Serum and sugar-free DMEM medium was used as hypoxic fluid replacement medium, and cardiomyocytes of Control, EV and COX6B1 groups were incubated in a closed hypoxic incubator (95% N_2_ and 5% CO_2_) at 37 °C for 8 h. 95% N_2_ and 5% CO_2_ mixed gas was vented at a flow rate of 2 L/min to replace the air in the incubator. After the cells had been hypoxed, fresh DMEM medium (with 0.5% FBS) was replaced and then cells were cultured at 37 °C with 5% CO_2_ for 16 h.

### Cell viability assay

Cell counting kit 8 (CCK8; Beyotime, Nantong, China) was performed to detect cell viabilities in Control, EV, COX6B1, I/R, I/R + EV and I/R + COX6B1 groups. Cells were seeded in a 96-well plate at the concentration of 5 × 10^3^ cells/well and cultured for 12 or 24 h. Then 20 μL CCK-8 reagent was added and incubated with the cells for 1 h. Optical density (OD) values were observed at 450 nm using a microplate reader (Thermo, USA).

### Cell apoptosis assay

Annexin-V/PI double-stain kit (Roche, Switzerland) was used to determine cell apoptosis in Control, EV, COX6B1, I/R, I/R + EV and I/R + COX6B1 groups. Cells were seeded in a 96-well plate at the concentration of 5 × 10^3^ cells/well, and stained by 5 μL Annexin-V and 5 μL PI in the dark for 5 min at room temperature. The analysis was immediately conducted using a flow cytometer (BD, San Diego, USA) and Cell Quest software.

### Mitochondrial membrane potential assay

Mitochondrial membrane potential assay kit with JC-1 (Beyotime, China) was used to determine mitochondrial membrane potential in Control, EV, COX6B1, I/R, I/R + EV and I/R + COX6B1 groups. Cells were cultured in 24-well plates at an initial density of 5 × 10^5^ cells/well and reacted with JC-1 probe (10 μmol/L reaction concentration) at 37 °C for 20 min. The analysis was conducted by a flow cytometer (BD, San Diego, USA) and Cell Quest software.

### Mitochondria isolation

Mitochondria were isolated from cardiomyocytes (in Control, EV, COX6B1, I/R, I/R + EV and I/R + COX6B1 groups) using Cell Mitochondria Isolation Kit (Beyotime, China). The cells were centrifuged at 4 °C, at 1500×*g*, for 10 min. The mitochondria-containing precipitate was then collected, and the supernatant was centrifuged at 4 °C, at 1000×*g* for 10 min in order to obtain cytoplasmic proteins with non-mitochondria. The protein content of mitochondria and cytoplasmic proteins were detected in a microplate reader (wavelength of 595 nm) using Bradford Protein Concentration Assay Kit (Beyotime, China).

### MPTP assay

MPTP colorimetric assay kit (Genome, China) was used for mPTP detection, and 20 μL mitochondrial samples (in Control, EV, COX6B1, I/R, I/R + EV and I/R + COX6B1 groups) were pipetted into the corresponding wells of a 96-well plate. After adding 170 μL buffer, cells were immediately placed in a microplate reader (wavelength of 540 nm). Then 10 μL inducing solution was added in, and the cells were immediately placed in the microplate reader (wavelength of 540 nm). Change at 18 min was obtained. The absorbance ratio (A540/Initial A540) was calculated to indicate mPTP opening condition.

### Western blot

Proteins, including Mitochondria cyto c, Cytosol cyto c (cytochrome c), COX, Pro-Caspase-3, Cleaved-Caspase-3, Pro-Caspase-9, Cleaved-Caspase-9, Bcl-2 and Bax, were detected using sodium dodecyl sulfate-polyacrylamide gel electrophoresis (SDS-PAGE), with all lanes being loaded with 20 μg protein/lane. Next, the separated proteins were electroblotted to a polyvinylidene fluoride (PVDF) membrane (Millipore, USA), which were blocked with 5% non-fat dry milk at 37 °C for 1 h. The membrane was probed with specific primary antibodies (Abcam, USA) overnight at 4 °Cand then with HRP-conjugated secondary antibodies (Abcam, USA). The immunoreactive bands were then detected by enhanced chemiluminescense (ECL) reagents (Millipore, USA). β-actin was used as loading control. Finally, the protein densities were quantified by densitometry (Bio-Rad, USA).

### Realtime-quantitative polymerase chain reaction (RT-qPCR)

The mRNA expression levels of apoptosis-related factors such as Bcl-2 (B cell lymphoma/leukmia-2) and Bax (Bcl-2 associated X protein) in Control, EV, COX6B1, I/R, I/R + EV and I/R + COX6B1 groups were determined by RT-PCR. First, total RNA was extracted using RNeasy kit (Qiagen, USA). 1 μg RNA was then reversely transcribed to cDNA using High-capacity cDNA Reverse Transcription Kit (Applied Biosystems, USA). Next, PCR amplification programs was performed at 95 °C for 30 s, 40 cycles (at 95 °C for 25 s; at 60 °C for 25 s; at 72 °C for 30 s) using Fast SYBR Green Master Mix (Applied Biosystems, USA) in ABI 7300 Thermocycler (Applied Biosystems, USA). The primer sequences were listed in Table 1.

**Table 1:** The primers applied in the study

Name	Type	Sequence (5′–3′)
β-actin	Forward	GTGGACATCCGCAAAGAC
	Reverse	GAAAGGGTGTAACGCAACT
COX6B1	Forward	AACTGCTGGCAGAACTACCTGG
	Reverse	AGAGGGACTGGTACACACGCTG
Bax	Forward	AACATGGAGCTGCAGAGGAT
	Reverse	CCAATGTCCAGCCCATGATG
Bcl-2	Forward	TTCTTTGAGTTCGGTGGGGT
	Reverse	CTTCAGAGACAGCCAGGAGA

### Statistical assay

Data were shown as mean ± standard deviations (mean ± SD). SPSS 18.0 statistical software was used for statistical analysis one-way analysis of variance (ANOVA) with Turkey test was used to compared the differences. P < 0.05 was considered as significantly difference. All experiments were repeated at least for three times.

## Results

### The cardiomyocytes of neonatal rats were isolated

**Fig. 1:**
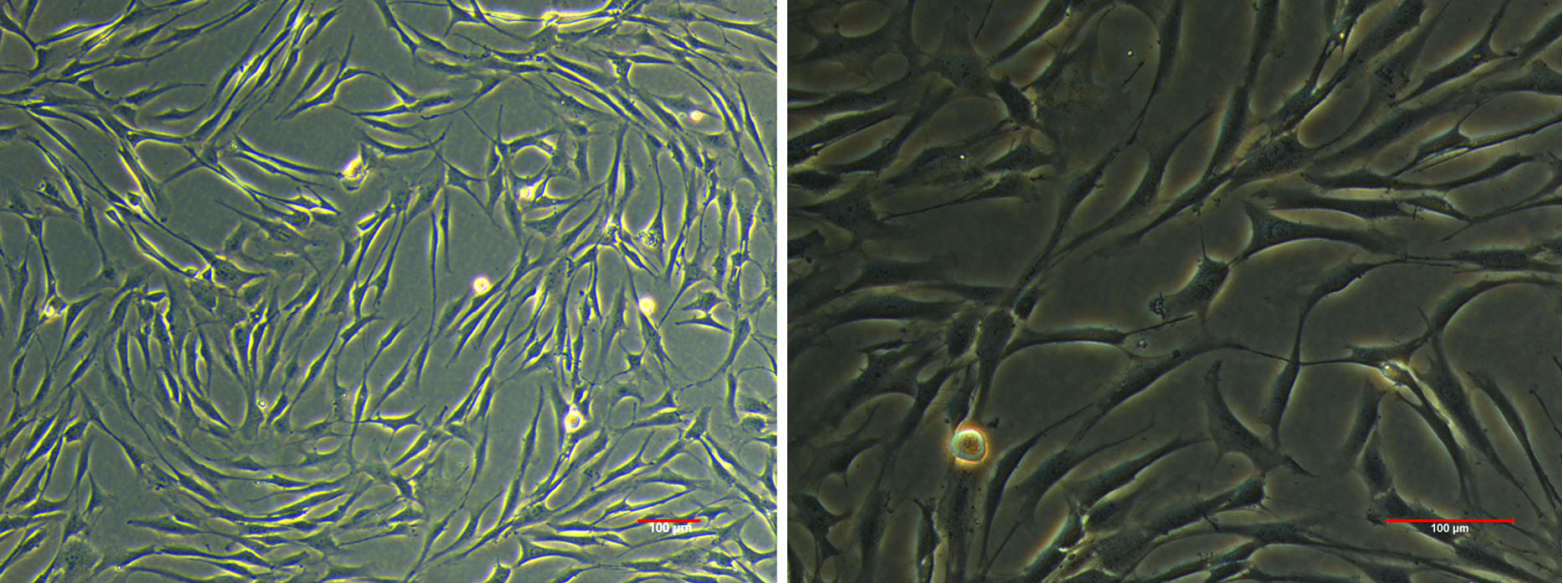
The cardiomyocytes of neonatal rats were isolated and authenticated under microscope with 200-fold and 400-fold magnification

The cardiomyocytes of neonatal rats, with certain proliferation ability, were isolated from the neonatal SD rats (within 24 h of birth) by repeated digestion of collagenase and trypsin. After being cultured for 48 h, the cardiomyocytes were observed under inverted microscope with 200-fold and 400-fold magnification. The results showed that some of the cardiomyocytes were adhered to the particulate-like rough substances (Fig. 1).

### COX6B1 was over-expressed in cardiomyocytes transfected with COX6B1

**Fig. 2:**
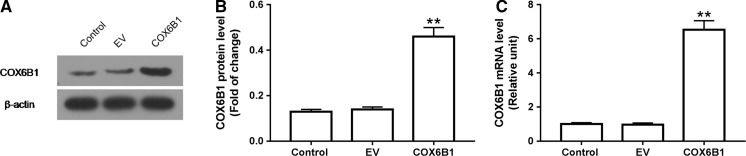
COX6B1 was over-expressed in cardiomyocytes transfected with COX6B1. **a**, **b** The protein level of COX6B1 was significantly up-regulated in COX6B1 group. **c** The mRNA expression of COX6B1 was significantly up-regulated in COX6B1 group. **P < 0.01 versus Control group

COX6B1 was transfected to cardiomytocytes of neonatal rats in order to construct COX6B1 over-expression cell model. By measuring the mRNA and protein levels of COX6B1, the transfection efficiency was detected using RT-qPCR and Western blot. The results demonstrated thatboth the mRNA and protein levels of COX6B1 were significantly up-regulated in COX6B1 group, compared with Control group (P < 0.01, Fig. 2).

### COX6B1 over-expression inhibited apoptosis and enhanced mitochondrial function in cardiomyocytes of neonatal rats with I/R

**Fig. 3:**
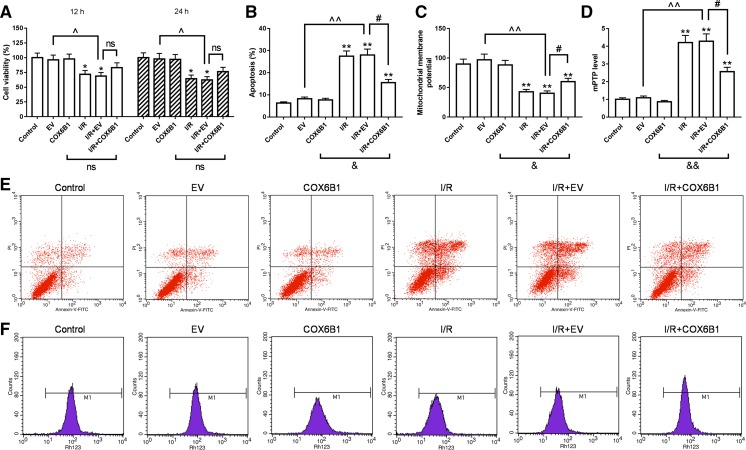
COX6B1 over-expression inhibited apoptosis and enhanced mitochondrial function in cardiomyocytes of neonatal rats with I/R. **a** Cell viability was slightly promoted by COX6B1 over-expression in I/R + COX6B1 group. **b** and **e** Apoptosis rates were significantly decreased by COX6B1 over-expression in I/R + COX6B1 group. **c** and **f** Mitochondria membrane potentials were significantly increased by COX6B1 over-expression in I/R + COX6B1 group. **d** MPTP was noticeably decreased by COX6B1 over-expression in I/R + COX6B1 group. *P < 0.05 and **P < 0.01 versus Control group, ^^^P < 0.05 and ^^^^P < 0.01 versus EV group, ^#^P < 0.05 versus I/R + EV group. ^&^P < 0.05 and ^&&^P < 0.01 versus COX6B1 group

I/R injury was created on cardiomyocytes of neonatal rats, and the cells were transfecdted with COX6B1 or empty vector. CCK-8 assay showed that I/R significantly inhibited cell viability of cardiomyocytes in I/R and I/R + EV groups at 12 and 24 h, and that cell viability was slightly promoted by COX6B1 over-expression compared to I/R injury (Fig. 3a). Apoptosis rates were significantly increased by I/R in I/R, I/R + EV and I/R + COX6B1 groups, however, COX6B1 over-expression significantly decrease the apoptosis rates in I/R + COX6B1 group, compared with I/R + EV group (Fig. 3b, e). Mitochondria membrane potentials were significantly decreased by I/R in I/R, I/R + EV and I/R + COX6B1 groups, by contrast, COX6B1 over-expression significantly increase the mitochondrial membrane potentials in I/R + COX6B1 group, compared with I/R + EV group (Fig. 3c, f). The mPTP levels were noticeably increased by I/R in I/R, I/R + EV and I/R + COX6B1 groups. However, COX6B1 over-expression significantly decrease the mPTP levels in I/R + COX6B1 group, compared with I/R + EV group (Fig. 3d).

### COX6B1 over-expression inhibited the release of cyto C from mitochondria to cytoplasm in cardiomyocytes of neonatal rats with I/R

**Fig. 4:**
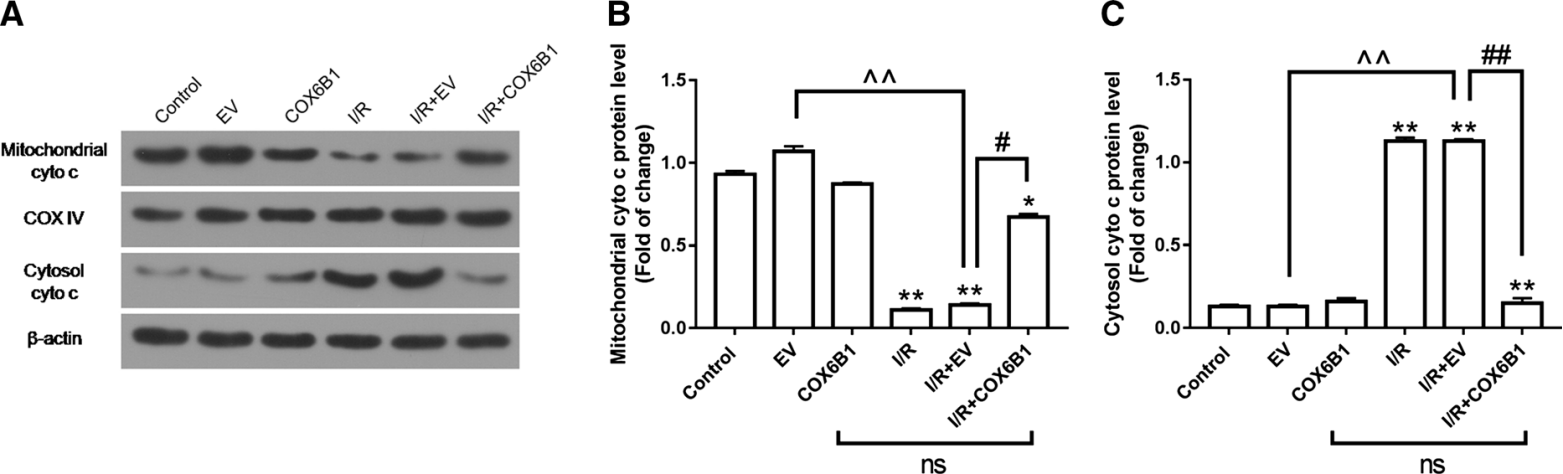
COX6B1 over-expression inhibited the release of cyto C from mitochondria to cytoplasm in cardiomyocytes of neonatal rats with I/R. **a**–**c** Mitochondrial cyto c was significantly promoted, and Cytosol cyto c was significantly inhibited by COX6B1 over-expression in I/R + COX6B1 group. COX served as an internal control for mitochondrial protein. **P < 0.01 versus Control group, ^^^^P < 0.01 versus EV group, ^##^P < 0.01 versus I/R + EV group

The protein levels of cyto C in mitochondria and cytoplasm, and COX were detected by carrying out Western blot in cardiomyocytes in neonatal rats. The results showed that the expression of mitochondrial cyto c was significantly inhibited while the expression of cytosol cyto c was increased in I/R in I/R and I/R + EV groups, however, as an internal control for mitochondrial protein, no significant change of COX IV levels among all detected groups was observed. COX6B1 over-expression promoted the expression of cytosol cyto c but inhibited the expression of mitochondrial cyto c compared to I/R and I/R + EV groups (Fig. 4).

### COX6B1 over-expression inhibited cell apoptosis by regulating apoptosis-related factors in cardiomyocytes of neonatal rats with I/R

**Fig. 5:**
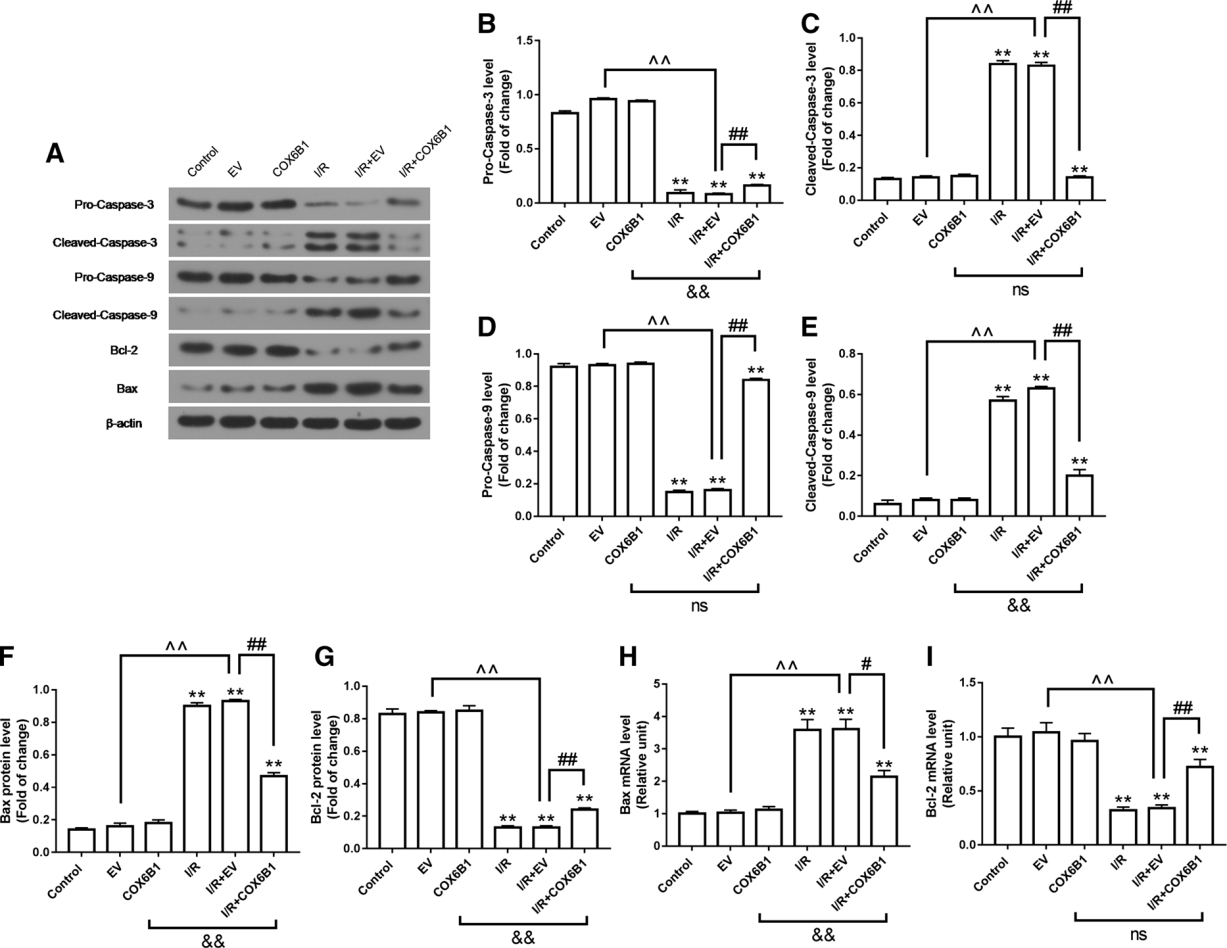
COX6B1 over-expression inhibited cell apoptosis by regulating apoptosis-related factors in cardiomyocytes of neonatal rats with I/R. (A-G) The protein levels of Cleaved-Caspase-3, Cleaved-Caspase-9 and Bax were significantly down-regulated, and levels of Pro-Caspase-3, Pro-Caspase-9 and Bcl-2 were significantly up-regulated by COX6B1 over-expression in I/R + COX6B1 group. (F and H) The Bax mRNA level was significantly down-regulated, and Bcl-2 mRNA level was significantly up-regulated by COX6B1 over-expression in I/R + COX6B1 group. **P < 0.01 versus Control group, ^^^^P < 0.01 versus EV group, ^#^P < 0.05 and ^##^P < 0.01 versus I/R + EV group. ^&&^P < 0.01 versus COX6B1 group

Data from Western blot data showed that the expression levels of activated Cleaved-Caspase-3 and Cleaved-Caspase-9 were noticeably down-regulated in I/R and I/R + EV groups, compared with I/R + COX6B1 group (Fig. 5a, c, e). Meanwhile, the expression levels of Pro-Caspase-3 and Pro-Caspase-9 were significantly down-regulated in I/R and I/R + EV groups, compared with Control group. By contrast, a significant up-regulation of the expression levels of Pro-Caspase-3 and Pro-Caspase-9 by COX6B1 over-expression was observed in I/R + COX6B1 group (Fig. 5a, b, d). In addition, both the mRNA and protein levels of Bax was significantly up-regulated in I/R and I/R + EV groups, compared with Control group, but significantly down-regulated by COX6B1 over-expression in I/R + COX6B1 group (Fig. 5a, f, h). Meanwhile, both the mRNA and protein levels of Bcl-2 were significantly down-regulated in I/R and I/R + EV groups, compared with Control group, but noticeably up-regulated by COX6B1 over-expression in I/R + COX6B1 group (Fig. 5a, g, i).

## Discussion

Cardiomyocytes are the most important functional cells in the heart (Geng et al. 2018). When being cultured in vitro, cardiomyocytes could maintain a variety of original cellular structures and functions, for example, spontaneous rhythm (Riching et al. 2018). This provides an excellent in vitro model for studying various molecular mechanisms of heart. Cardiomyocyte culture has become a basic method for investigating cardiac physiology and pathology, and it provides an ideal model for the study of heart development, differentiation, and metabolism (Boogerd et al. 2018). Therefore, the cultivation of cardiomyocytes has gradually become a useful model for studying myocardial injury (Liu et al. 2018). In this study, one-day-old neonatal rats were selected for the research, and the cardiomyocytes had strong reproductive abilities. Primary cultured neonatal rat cardiomyocytes were successfully obtained by continuous digestion of trypsin and collagenase, and a myocardial hypoxia/reoxygenation model was established to simulate an injury environment of cardiomyocytes after I/R injury. Our data confirmed that COX6B1 overexpression could significantly reduce the apoptosis rates of cardiomyocytes after hypoxia/reoxygenation, and that it had a significant mitochondrial protective effect, which protected cardiomyocytes from hypoxia/reoxygenation injury.

A large number of studies have confirmed that ischemia and hypoxia not only lead to necrosis, but also induce apoptosis of cardiomyocytes. Apoptosis has a profound effect on the myocardium, and inhibition of apoptosis can relieve myocardial damage (Breglia et al. 2018; Wang et al. 2018a). Our results show that over-expression of COX6B1 could effectively restrain cell apoptosis during myocardial ischemia. Apoptosis is a type of programmed cell death that is regulated by a series of genes (El-Gamal and Gouida 2013). Among those genes, Bcl-2 gene is the first identified apoptosis-inhibiting gene (Pan et al. 2014). Bcl-2 protein is mainly distributed in the inner mitochondrial membrane, and its high expression can inhibit apoptosis caused by a variety of factors (Bittremieux et al. 2018). Bax is the most important apoptosis-promoting factor in Bcl-2 family (Wang et al. 2018b). Mitochondrial pathway is alternatively known as endogenous apoptotic pathway, and it is one of the most important pathways for apoptosis (Wang et al. 2015a). Massive opening of mPTP is a primary cause that induces cell apoptosis (Fakharnia et al. 2017). By regulating the opening of mPTP, Bcl-2 family proteins are the key regulatory factors of this pathway (Chen et al. 2015). Our study found that in the hypoxia-reoxygenation model of myocardiocytes, mPTP was mostly opened and could laed to the disruption of mitochondrial outer membrane. Therefore, mitochondrial membrane potential was reduced, leading to the release of pro-apoptotic factors, such as cytochrome c, from the mitochondrial intermembrane space. The concentration of cytochrome C in the cytoplasm increased significantly, while cytochrome C in the mitochondria decreased significantly. The extravasated cytochrome c activates Caspase-9, which in turn cleaves Caspase-3 to be its active subunit (Wen et al. 2014). Activated Caspase-3 has an ability of cleaving peptide bonds after an aspartate residue, and this would result in the degradation and inactivation of intracellular important proteins, DNA fragmentation, and apoptosis of cardiomyocytes (Zhao et al. 2014). Overexpression of COX6B1 significantly reduced the opening of mPTP, the disruption of mitochondrial outer membranes, and cytoplasmic cytochrome C levels in hypoxic and reoxygenated cardiomyocytes. Meanwhile, the levels of Bax, Cleaved-caspase-3, and Cleaved-caspase-9 were decreased and Bcl-2 level was increased, and such phenomena inhibited the apoptosis of cardiomyocytes subjected to hypoxia-reoxygenation.

Myocardial cells cannot adapt quickly to hyperoxia caused by a sudden restoration of blood flow after continuous ischemia. Thus, the myocardial cells are damaged. Overexpression of COX6B1 not only inhibits mPTP opening, cell apoptosis and the mitochondrial membrane rupture of cardiomyocytes, but also reduces the release of cytochrome C. Therefore, overexpression of COX6B1 plays a critical role in myocardial protection.
